# Direct and indirect effects of childhood adversity on psychopathology: Investigating parallel mediation via self‐concept clarity, self‐esteem and intolerance of uncertainty

**DOI:** 10.1111/bjc.12523

**Published:** 2024-12-08

**Authors:** Lindsey Sharratt, Nathan Ridout

**Affiliations:** ^1^ School of Psychology, College of Health and Life Sciences Aston University Birmingham UK

**Keywords:** early adversity, hypomania, identity‐disruption‐model, personal identity, psychopathology

## Abstract

**Objectives:**

The aim was to extend previous work on the identity disruption model (IDM) of adult psychological distress. According to the IDM, aversive childhood experiences (ACEs) disrupt the development of identity, resulting in an unclear sense of self and a reliance on external sources of self‐definition, leading to psychological distress in adulthood. In line with this model, self‐concept clarity (SCC) in parallel with self‐esteem (SE) and intolerance of uncertainty (IU) has been shown to mediate the relationship between childhood adversity and depression and anxiety. The current study examined if SCC, SE and IU mediated the influence of childhood adversity on depression, anxiety and hypomania.

**Methods:**

A community sample of 159 adults completed online measures of childhood adversity, self‐esteem, self‐concept clarity, intolerance of uncertainty, depression, anxiety and hypomania. Structured equation modelling using bias corrected bootstrapping was used to test the mediation model.

**Results:**

Direct effects of childhood adversity were found for depression and anxiety, but not hypomania. The influence of ACEs on depression and anxiety was mediated by self‐concept clarity and self‐esteem. Self‐concept clarity also mediated the influence of ACEs on hypomania, which is an important novel finding. The indirect effect of childhood adversity via intolerance of uncertainty was limited to anxiety.

**Conclusions:**

Results suggest that the identity disruption model generalizes to hypomania. The clinical implications are that interventions to improve clarity of the self‐concept might be useful in reducing psychopathology.

**Practitioner points**

- Self‐concept clarity and self‐esteem mediated the influence of childhood adversity on depression and anxiety.
- Self‐concept clarity also mediated the relationship between childhood adversity and hypomania.
- Targeted interventions to improve the clarity of individuals' self‐concept has the potential to improve psychological well‐being.

## INTRODUCTION

Aversive childhood experiences (ACEs), such as abuse, neglect, or unstable home‐life, have a negative impact on physical and psychological wellbeing that endures into adult life (Hughes et al., 2017; Kessler et al., 2010; Martins et al., 2011). For example, ACEs have been shown to predict depression and other psychopathology (e.g. substance abuse) in adulthood (Afifi et al., 2009; Infurna et al., 2016). Adversity in childhood is thought to alter the development and function of the brain, endocrine and immune systems, which is then linked to poor physical and mental health (Boullier & Blair, 2018). However, work has also begun to explore the psychological mechanisms that might contribute to the link between childhood adversity and psychological distress in adulthood. One avenue has examined the possible role of the self. In particular, two important concepts have been examined in the context of childhood adversity and adult psychopathology: self‐identity and self‐concept. Self‐identity refers to the traits and characteristics, social relations, roles and social group memberships that define who one is (Oyserman, 2001). Self‐concept refers to beliefs an individual holds about their abilities, behaviour and defining characteristics (Harter & Leahy, 2001). One model that has emerged from this work is the identity disruption model (IDM; Vartanian et al., 2018). The IDM was initially developed to explain the link between childhood adversity and disordered eating, with the key proposition being that negative experiences in childhood disrupt the development of identity, leading to an unclear sense of self, which in turn results in a greater reliance on external sources of self‐definition. In the context of disordered eating, unstable self‐identity as a result of childhood adversity, leads to increased social comparison and internalization of cultural beliefs about beauty, which in turn leads to increased body dissatisfaction and disordered eating (Vartanian et al., 2018). This model has subsequently been used to account for the link between childhood adversity and other psychopathologies, notably depression and anxiety (Hayward et al., 2020; Wong et al., 2019). These studies identified three important psychological factors that contribute to the relationship between childhood adversity and psychopathology: self‐concept clarity, self‐esteem and intolerance of uncertainty.

Self‐concept clarity refers to ‘the degree to which the self‐concept is clearly and confidently defined, temporally stable and internally consistent’ (Campbell et al., 1996, p.141). This factor is lower in participants who have experienced childhood adversity (Streamer & Seery, 2015; Vartanian et al., 2018) and has been linked to various psychopathologies, including depression, anxiety and suicidal behaviour (Butzer & Kuiper, 2006; Cicero, 2018; Hayward et al., 2020; Wong et al., 2019). Importantly, self‐concept clarity has been shown to mediate the influence of childhood adversity on depression and anxiety (Hayward et al., 2020; Wong et al., 2019), which supports the identity disruption model.

Self‐esteem, which is the degree to which one's attitude to oneself is favourable or unfavourable (Rosenberg, 1965), has been implicated in the development of psychopathology. For example, a meta‐analysis confirmed it is strongly related to depression and anxiety (Sowislo & Orth, 2013). Self‐esteem has also been linked with childhood adversity. For example, Shen (2009) reported lower self‐esteem in young adults who had experienced maltreatment as children. Furthermore, self‐esteem has been shown to mediate the link between adverse childhood experiences and depression (Kim & Cicchetti, 2006; Kim et al., 2022; Wong et al., 2019) and other mental health conditions (Chartier et al., 2009). Self‐esteem is strongly linked to self‐concept clarity (Campbell, 1990) but the exact nature of this relationship is unclear. It has been proposed that self‐esteem might precede self‐concept clarity (Wu, 2009). However, there is some contradictory evidence, for example self‐concept clarity is also thought to be a pillar of stable self‐esteem (Kernis et al., 2000), which is important because individuals with unstable self‐esteem are more emotionally reactive to negative life events and exhibit greater depression and anxiety in response to daily hassles (Kernis, 2005). In adulthood, the relationship between self‐esteem and self‐concept clarity is thought to be reciprocal (Campbell, 1990). Importantly, Wong et al. (2019) demonstrated self‐esteem and self‐concept clarity were parallel mediators of the relationship between childhood adversity and adult mental health outcomes.

An additional factor thought to play a role in the susceptibility to external factors (e.g. adverse childhood experiences) and the development of psychopathology is intolerance of uncertainty (Freeston et al., 1994), which manifests as a desire for predictability, certainty seeking and cognitive paralysis in the face of uncertainty. Intolerance of uncertainty is a risk factor for generalized anxiety disorder (Birrell et al., 2011), but has also been linked to other psychopathologies, such as depression, social anxiety and obsessive‐compulsive disorder (Butzer & Kuiper, 2006; Hayward et al., 2020). Intolerance of uncertainty has also been shown to predict the frequency of social comparisons (Butzer & Kuiper, 2006), which is important given that social comparison is an integral part of the identity disruption model (Vartanian et al., 2018). As adverse childhood experiences are chaotic, unpredictable and uncontrollable it plausible that these events might result in intolerance of uncertainty (Hayward et al., 2020), which in turn would be expected to predict depression and anxiety. Given the evidence of a negative relationship between intolerance of uncertainty and self‐concept clarity (Kusec et al., 2016); Hayward et al. (2020) examined the combined mediating effects of these factors on the link between childhood adversity and psychopathology, and provided evidence of parallel mediation for depression, anxiety, OCD and social anxiety. It is notable that intolerance of uncertainty is also negatively related to self‐esteem (Lowe & Harris, 2019), which suggests that it might also affect the mediating influence of self‐esteem on the link between adverse childhood experiences and adult mental health.

Given the above, work to further elucidate the role of self‐esteem and intolerance of uncertainty in the link between childhood adversity and adult psychopathology, particularly in the context of self‐concept clarity and the identity disruption model, is an important avenue of research. As intolerance of uncertainty is linked to both self‐concept clarity (Wong et al., 2019) and self‐esteem (Lowe & Harris, 2019) it is important to establish if the findings in depression and anxiety (Hayward et al., 2020; Wong et al., 2019) hold in a model with all three factors as parallel mediators. Furthermore, as childhood adversity predicts hypomanic symptoms (Johnson et al., 2015), an important question is whether self‐concept clarity, self‐esteem and/or intolerance of uncertainty would also mediate this relationship. In regard to the possible role of self‐concept clarity in hypomania, unstable self‐identity might result in the individual focusing on external events that provide opportunities for goal pursuit and goal success, in order to bolster their sense of self. In line with this proposal, evidence supports the link between goal pursuit and goal attainment events and manic/hypomanic symptoms (Nusslock et al., 2007; Proudfoot et al., 2011). The relationship between hypomania and self‐esteem is complex with some studies showing low self‐esteem in individuals with hypomania (Pavlickova et al., 2013) and bipolar disorder (Pavlova et al., 2011) and others showing no difference between manic and healthy controls (Park et al., 2014). Self‐esteem in bipolar patients appears to be contingent on goal achievement (Ironside et al., 2020). In line with this proposal, Pavlova et al. (2011) manipulated success and failure using an anagram test and showed that patients with bipolar disorder showed fluctuations in their self‐esteem that was consistent with their success or failure on the task. Importantly, childhood trauma was linked to less stable self‐esteem in bipolar patients. To date, we are unaware of any proposed link between intolerance of uncertainty and hypomania. However, McGregor et al. (2010) demonstrated that anxious uncertainty was linked to reactive approach motivation, which is a form of goal directed behaviour, in an attempt to distract from this uncertainty. As adverse childhood events are unpredictable and uncontrollable they might lead to uncertainty (about the world and the self), which in turn could lead to reactive approach motivation and, in certain individuals, hypomania. This is also consistent with the behavioural approach system (BAS) dysregulation model of bipolar disorder (Depue & Iacono, 1989). The BAS refers an engagement system that facilitates behaviour related to goal seeking and reward. According to the BAS dysregulation model, manic/hypomanic behaviour is linked to an overactive BAS system with goal attainment experiences leading to increased positive affect and anger‐inducing events leading to irritability. An alternative but complementary explanation is the manic defence hypothesis of bipolar disorder (Lyon et al., 1999; Winters & Neale, 1985), which suggests that individuals engage in hypomanic behaviour (e.g. grandiose thinking & goal seeking) to defend against feelings of worthlessness (low self‐esteem) arising from underlying negative self‐schema (Carlstedt, 2009), which are linked to childhood adversity (Lumley & Harkness, 2007). Taken together, it is plausible that self‐concept clarity, self‐esteem and intolerance of uncertainty would mediate the link between childhood adversity and hypomania.

The current aims were (a) to establish if findings concerning the direct and indirect effects of childhood adversity on depression and anxiety hold when self‐concept clarity, self‐esteem and intolerance of uncertainty are all included as parallel mediators and (b) to determine if these factors mediate the link between ACEs and hypomania. To this end, a community sample of participants completed online measures of self‐concept clarity, self‐esteem, intolerance of uncertainty, aversive experiences in childhood and symptoms of psychopathology (depression, anxiety and hypomania). Based on Wong et al. (2019) and Hayward et al. (2020) it was expected childhood adversity would directly predict depression and anxiety. Similarly, based on Johnson et al. (2015), it was expected that childhood adversity would directly predict hypomanic symptoms. Furthermore, it was expected that childhood adversity would predict self‐concept clarity (Hayward et al., 2020; Wong et al., 2019), self‐esteem (Wong et al., 2019) and intolerance of uncertainty (Hayward et al., 2020), which in turn would predict depression and anxiety. Finally, as childhood adversity is expected to lead to uncertainty (about the world and the self) and to undermine self‐esteem, which in turn might lead to reactive approach motivation and goal seeking behaviour then it is plausible that self‐concept clarity, self‐esteem and intolerance of uncertainty would mediate the link between childhood adversity and hypomania.

## METHOD

### Design

The current study used a cross‐sectional questionnaire design. The predictor variable was childhood adversity, the dependent variables were depression, anxiety and hypomania. The mediator variables were self‐concept clarity, self‐esteem and intolerance of uncertainty.

### Participants

A community sample of 186 participants was recruited via Prolific (www.prolific.com), which is an online research platform that enables rapid and reliable data collection from participants from diverse backgrounds across the world and social media (Facebook, X (formerly Twitter)). Inclusion criteria included being aged between 18 and 65 and being able to read and understand written English. The data from 27 participants (15% of the sample) were excluded from the analysis due incomplete or missing measures. Therefore, the data from 159 participants (30 males, 127 females; mean age = 41.8 years, SD = 12.0) were included in the structured equation model. All participants provided informed consent and the study was approved by Aston University's Research Ethics Committee.

### Materials and measures

*Self‐Concept Clarity Scale* (*SCC*) (Campbell et al., 1996): The SCC is a 12‐item scale that measures coherence, stability and definition of the sense of self (e.g. “In general, I have a clear sense of who I am and what I am”). Participants rate each item from 1 (strongly disagree) to 5 (strongly agree). The range of scores on this measure is 12–60, with higher scores indicating greater clarity of self‐concept. This scale has been used in previous studies examining the link between childhood adversity and psychopathology (Wong et al., 2019). The SCC has good validity and reliability Campbell et al. (1996) and SCC scores in the current study showed excellent internal consistency, Cronbach's alpha = .92.

*Rosenburg Self‐Esteem Scale* (*SES*) (Rosenberg, 1965): The SES is a 10‐item scale assessing global self‐worth (e.g. ‘I feel that I'm a person of worth, at least on an equal plane with others’). Participants rate each item on a 4‐point scale from strongly disagree to strongly agree. Each item is scored 0–4 (negative items are reverse scored) and the range of scores on the measure is 0–40, with higher scores indicating more positive self‐esteem. This measure has been frequently used in self‐esteem studies (Ekinci & Kandemir, 2015). The SES is a reliable (Greenberger et al., 2003) and SES in the current study showed excellent reliability, Cronbach's alpha = .93.

*Intolerance of Uncertainty Scale Short Version* (*IUS‐12*) (Carleton et al., 2007): The IUS‐12 is a 12‐item scale measuring reactions to uncertainty, ambiguity and future situations (e.g. ‘When I am uncertain, I can't function very well’). Participants rate each item on a scale from 1 ‘Not at all characteristic of me’ to 5 ‘Entirely characteristic of me’. The range of scores is 12–60, with higher scores indicating greater intolerance of uncertainty. This scale has been used in previous studies examining the link between ACEs and psychopathology (Hayward et al., 2020). The IUS‐12 is reliable (Carleton et al., 2007) and IUS‐12 scores in the current study showed a high internal consistency, Cronbach's alpha = .92.

*Adverse Childhood Experiences* (*ACE Scale*) (Felitti et al., 1998): The ACE is a 10‐item scale measuring three categories of adverse childhood experiences: abuse (emotional, physical, sexual); neglect (physical & emotional) and household challenges (e.g. violence, drug abuse, divorce). Participants respond either ‘yes’ or ‘no’ depending on whether they experienced each adversity before their 18th birthday. The number of ‘yes’ responses is summed, so the range of possible scores is 0–10, with higher score indicating greater childhood adversity. This scale has been used in other studies looking at the link between ACEs and psychopathology (Wong et al., 2019). This measure is reliable (Murphy et al., 2014), the Cronbach's alpha for the current ACE scores was .77, indicating good reliability.

*Risky Families Questionnaire* (*RFQ*) (Taylor et al., 2004): The RFQ is an 11‐item scale measuring participants' perceptions of growing up in a stressful and dysfunctional household between the ages of 5 and 15 (e.g. ‘How often did a parent or other adult in the household swear at you, insult you, put you down, or act in a way that made you feel threatened?’) Each item was rated on a scale from 0 ‘Not at all’ to 4 ‘Very often’ (positive items are reverse scored), giving a range of possible scores of 0–44, with higher scores indicate greater family adversity. This scale has been used in other studies looking at the link between ACE and psychopathology (Hayward et al., 2020). The RFQ is a reliable measure (Coelho et al., 2014), which was confirmed in the current study, Cronbach's alpha = .90.

*Maryland Trait and State Depression Scale* (MTSD) (Chiappelli et al., 2014): The MTSD consists of two 18‐item scales measuring current ‘state’ and dispositional ‘trait’ depression. Participants rate how often they have experienced certain feelings or behaviours in the past week (e.g. ‘I cried because my mood was low’) or generally in their life. Each item is scored from 0 to 4 (positive items are reverse scored), thus the range of possible scores on each subscale (trait and state) is 0–72, with higher scores equating to more severe depression. This measure has been shown to be reliable (Chiappelli et al., 2014), which was confirmed in the current study; state depression (Cronbach's alpha = .95) and trait depression (.96).

*State–Trait Anxiety Inventory* (*STAI*) (Spielberger, 1983): The STAI consists of two 20‐item scales concerning situational ‘state’ and dispositional ‘trait’ anxiety. Participants are asked to indicate how they feel (e.g. ‘I am calm.’) at the time (state) and in general (trait). Each item is scored on a scale between 0 ‘not al all’ and 4 ‘Very much so’ (positive items are reverse scored) resulting in a range positive scores on each subscale of 20–80, with higher scores equating to greater anxiety. Previous studies have shown the measure to be reliable (Barnes et al., 2002), which was confirmed in the current study, with a Cronbach's alpha of .95 for both (state and trait) scales.

*Hypomanic Checklist* (*HCL‐32*) (Angst et al., 2005): The HCL‐32 is a 32‐item checklist in which participants report either ‘yes’ or ‘no’ to questions (e.g. ‘I think faster’) about certain behaviours that they carry out while in elevated mood. The measure is scored by summing the number of yes responses, with a range of possible scores being 0–32, with higher scores equating to greater number of hypomanic traits. A score on the HCL‐32 of 14 or above is considered indicative of clinically significant hypomanic traits. Previous work has confirmed that this measure provides good test–retest reliability (Angst et al., 2005), which was confirmed in the current study with a Cronbach's alpha of .86.

### Procedure

Participants were provided with an information sheet prior to giving informed consent. They then completed all measures online via Qualtrics. Measures were presented in the following order for all participants: SCCS, SES, IUS‐12, ACE, RFQ, MSTD‐S, MSTD‐T, STAI‐S, STAI‐T, HCL‐32.

### Scoring and data analysis

The data from 159 participants were analysed using structured equation modelling (maximum likelihood estimation) utilizing the syntax function in the *SEMLj* module in Jamovi (version 2.3.21). Adverse childhood experiences and risky families questionnaires were specified to load onto a latent variable, childhood adversity. As depression and anxiety were each assessed using two measures, these were specified to load onto latent variables of depression and anxiety. Hypomania was entered into the model as a measured variable, as were self‐concept clarity, self‐esteem and intolerance of uncertainty, which were specified as parallel mediators of the pathway between childhood adversity and the mental health outcomes. Bias corrected bootstrapping (5000 iterations) was used to estimate the 95% confidence intervals around the coefficients for the indirect effects of childhood adversity on depression, anxiety and hypomania (via self‐concept clarity, self‐esteem and intolerance of uncertainty). A good model fit is usually indicated by a non‐significant *χ* ^2^ test, an RMSEA of < .08, SRMR close to .08. Further, the comparative fit index (CFI) and Tucker‐Lewis Index (TLI) should be .95 or above (Hu & Bentler, 1999).

## RESULTS

The mean scores and standard deviations for each scale are shown in Table 1. This table also presents the bivariate correlations between the different measures.

**TABLE 1:** Means, standard deviations and bivariate correlations for the different measures.

Variable	Mean (SD)	2	3	4	5	6	7	8	9	10
State depression	22.35 (17.3)	.80***	.78***	.86***	.34***	−.64***	−.71***	.57***	.28***	.34***
2 Trait depression	27.01 (16.8)	‐	.62***	.82***	.37***	−.65**	−.70***	.58***	.35***	.36***
3 State anxiety	41.26 (13.4)		‐	.79***	.28***	−.61***	−.67***	.48***	.30***	.37***
4 Trait anxiety	44.57 (13.2)			‐	.40***	−.74***	−.79***	.68***	.32***	.34***
5 Hypomania	12.68 (5.9)				‐	−.39***	−.31***	.23**	.27***	.17*
6 SSC	37.09 (10.4)					‐	.78***	−.49***	−.27***	−.27***
7 Self‐esteem	17.55 (6.0)						‐	−.61***	−.27***	−.27***
8 IUS	34.34 (10.6)							‐	.24**	.23**
9 ACE	2.82 (2.4)								‐	.58***
10 RFQ	15.19 (10.1)									‐

Abbreviations: ACE, Adverse childhood experiences scale; IUS, intolerance of uncertainty scale; RFQ, Risky families questionnaire; SSC, self‐concept clarity.

* *p* < .05;

** *p* < .01;

*** *p* < .001.

### Direct and indirect pathways between childhood adversity and psychopathology

**FIGURE 1:**
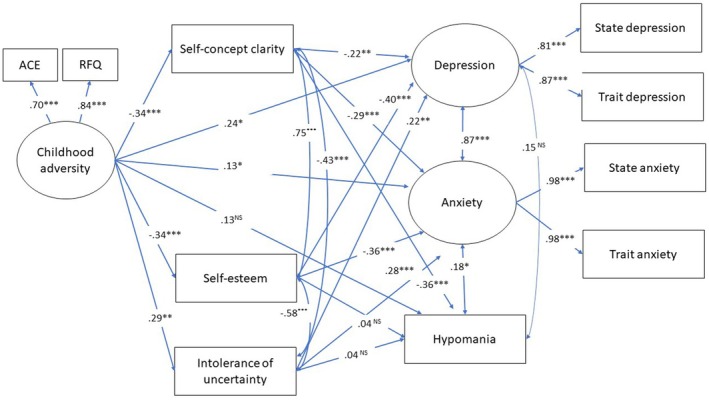
Structured equation model of childhood adversity predicting depression, anxiety and hypomania via the parallel mediators of self‐concept clarity, self‐esteem and intolerance of uncertainty. Standardized regression weights are reported. *p < .05, **p < .01 and ***p < .001.

The chi‐squared test (*χ* ^2^ (18) =51, *p* < .001) and RMSEA (.11, *p* = .004) suggests the model (shown in Figure 1) was not a great fit for the data. On the other hand, the SRMR (.03) and the fit statistics CFI (.97) and TTI (.93) suggest that model is a good fit to the data. Taken together the metrics suggest that the model is a reasonable fit to the data.

The overall model explained 74% of the variance in depression, 77% of the variance in anxiety and 17% of the variance in hypomania scores. There were significant direct effects of childhood adversity on depression and anxiety, but not hypomania. The indirect paths from childhood adversity to depression, anxiety and hypomania via self‐concept clarity were all significant (see Table 2). The indirect effect of childhood adversity via self‐esteem was significant for depression and anxiety, but not hypomania. The indirect path from childhood adversity via intolerance of uncertainty was significant for anxiety but not depression or hypomania.

**TABLE 2:** Unstandardized direct and indirect effects (separately for self‐concept clarity, self‐esteem and intolerance of uncertainty) of childhood adversity on depression, anxiety and hypomania.

Outcome variable	*β*	SE	LLCI	ULCI	*p*
Direct effects					
Depression	2.24	.92	.56	4.32	.**014**
Anxiety	.83	.42	.12	1.87	.**047**
Hypomania	.46	.39	−.36	1.21	.24
Indirect effects (SCC)					
Depression	.67	.33	.19	1.58	.**043**
Anxiety	.62	.24	.26	1.29	.**009**
Hypomania	.42	.20	.13	.96	.**034**
Indirect effect (SE)					
Depression	1.25	.51	.43	2.55	.**015**
Anxiety	.79	.34	.28	1.68	.**018**
Hypomania	−.05	.16	−.42	.23	.77
Indirect effects (IU)					
Depression	.59	.30	.17	1.50	.051
Anxiety	.52	.23	.18	1.17	.**025**
Hypomania	.05	.10	−1.02	.30	.63

Abbreviations: IU, intolerance of uncertainty; SCC, self‐concept clarity, SE, self‐esteem.

## DISCUSSION

The link between adverse childhood experiences (ACEs) and psychopathology later in life is well established, but the psychological mechanisms by which negative experiences in childhood influence adult mental health are less well understood. Previous work has examined the utility of the identity disruption model (Vartanian et al., 2018) in explaining the link between childhood adversity and eating disorders and more recently depression and anxiety (Hayward et al., 2020; Wong et al., 2019). The identity disruption model proposes that adverse childhood experiences disrupt the development of self‐identity, leading to an unclear sense of self (poor self‐concept clarity), which in turn results in a greater reliance on external sources of self‐definition. Recent work has confirmed that self‐esteem (Wong et al., 2019) and intolerance of uncertainty (Hayward et al., 2020) are parallel mediators of the influence of childhood adversity on mental health outcomes (depression & anxiety), along with self‐concept clarity. Work to further elucidate the role of self‐esteem and intolerance of uncertainty in the link between childhood adversity and adult psychopathology, particularly in the context of self‐concept clarity and the identity disruption model, is an important avenue of research. Thus, the aims of the current study were (a) to establish if findings concerning the direct and indirect effects of childhood adversity on depression and anxiety hold when self‐concept clarity, self‐esteem and intolerance of uncertainty are all included as parallel mediators and (b) to determine if self‐concept clarity, self‐esteem and/or intolerance of uncertainty mediate the link between childhood adversity and hypomania.

As expected, there was a significant direct effect of childhood adversity on depression and anxiety, which is consistent with previous findings (Hayward et al., 2020; Wong et al., 2019). Also as expected, self‐concept clarity and self‐esteem mediated the effect of childhood adversity on depression and anxiety. Intolerance of uncertainty mediated the influence of childhood adversity on anxiety, as expected, but not depression. The indirect path from childhood adversity to depression and anxiety via self‐concept clarity is consistent with previous findings (Hayward et al., 2020; Wong et al., 2019) and supports the identity disruption model (IDM; Vartanian et al., 2018). It is plausible that depression and anxiety result from the influence of self‐concept clarity on how individuals respond to events in their lives. For example, low self‐concept clarity has been linked to poor coping (Kakounda‐Moullem & Israelashvili, 2020; Smith et al., 1996). In particular, it has been linked to greater rumination (Willis & Burnett, 2016), which in turn predicts depression (Nolen‐Hoeksema et al., 2008) and anxiety (Olatunji et al., 2013). Psychological distress might also arise from how individuals with low self‐concept clarity interact with others. For example, self‐concept clarity predicts relationship quality (Parise et al., 2019), is linked to reduced proactive problem‐solving in social contexts (Bechtoldt et al., 2010) and less intimacy in relationships (Lewandowski Jr et al., 2010). This is notable, as lower perceived social support is a key factor in the development of depression and anxiety (Scardera et al., 2020; Zhou et al., 2013). Poor self‐concept clarity has also been linked to greater worrying (Campbell et al., 2003), which is particularly relevant to anxiety.

The finding that self‐esteem mediated the link between adverse childhood experiences and depression is consistent with the Wong et al. (2019), but our findings confirm that this mediation was also evident for anxiety. These findings are congruent with cognitive theories of depression and anxiety (e.g. Beck et al., 1987), as negative thoughts about the self are thought to be a key risk factor for depression and have also been implicated in anxiety (Sowislo & Orth, 2013).

The evidence of an indirect pathway from childhood adversity to anxiety via intolerance of uncertainty is consistent with Hayward et al. (2020). It is plausible that childhood adversity, due to its unpredictable and uncontrollable nature, leads to intolerance of uncertainty, which in turn encouraged pathological worrying—a cardinal feature of anxiety (Gentes & Ruscio, 2011). However, our finding that this indirect pathway was not present for depression is inconsistent with the findings of Hayward et al. (2020). It is consistent with previous research reporting that intolerance of uncertainty was more strongly related to anxiety than depression (Jensen et al., 2016). Nevertheless, it is worth noting that the mediation effect for depression in our study approached conventional significance (*p* = .051), suggesting further work to examine the role of this factor in the link between childhood adversity and depression is warranted.

An important novel aspect of the current work was the exploration of the potential pathways from childhood adversity to hypomania. We found no evidence of a direct pathway from negative childhood events to hypomania, which contrasts with the findings of Johnson et al. (2015). Interestingly, neither self‐esteem nor intolerance of uncertainty mediated the influence of negative childhood experiences on hypomania. However, childhood adversity did influence hypomania indirectly via self‐concept clarity, which suggests that the identity disruption model generalizes to hypomania.

The key proposition of the identity disruption model is that negative experiences in childhood disrupt the development of identity, leading to an unclear sense of self (poor self‐concept clarity), which in turn results in a greater reliance on external sources of self‐definition. In the context of hypomania, an unclear sense of self‐identity might result in the individual focusing on external events that provide opportunities for goal pursuit and goal success, in order to bolster their sense of self. In line with this proposal, manic/ hypomanic symptoms have been linked to goal pursuit and goal attainment events (Nusslock et al., 2007; Proudfoot et al., 2011). Hypomania is also associated with elevated expectations of goal achievement (Urošević et al., 2008). However, hypomanic symptoms have also been linked to anger inducing events (Harmon‐Jones et al., 2002). These findings are also consistent with the behavioural approach system (BAS) dysregulation model of bipolar disorder (Depue & Iacono, 1989). The BAS refers an engagement system that facilitates behaviour related to goal seeking and reward. According to the BAS dysregulation model, manic/hypomanic behaviour is linked to an overactive BAS system, with goal attainment experiences leading to increased positive affect and anger‐inducing events leading to irritability. An alternative but complementary explanation is the manic defence hypothesis of bipolar disorder (Lyon et al., 1999; Winters & Neale, 1985), which suggests that individuals engage in hypomanic behaviour (e.g. grandiose thinking & goal seeking) to defend against feelings of worthlessness (low self‐esteem) arising from underlying negative self‐schema (Carlstedt, 2009), which are linked to childhood adversity (Lumley & Harkness, 2007). However, the manic defence hypothesis would predict a mediating effect of self‐esteem, which was not observed in the current study.

### Limitations and future directions

The main limitation is that the current data are correlational and cross‐sectional, and therefore limited in the extent to which it can show causal pathways. Nevertheless, the mediation analyses were based on models that have logical conceptual timing between the variables, which is vital for the validity of cross sectional mediation models (Tate, 2015). It would be beneficial to carry out longitudinal studies to examine the time ordered relationships between variables and also to examine the influence of self‐concept clarity (and the other mediators) on the relationship between recent aversive events and psychopathology. The sample size was also relatively small to conduct a structured equation model. Results implicating self‐concept clarity in hypomania are novel and interesting but require further research. Studies could explore the impact of low‐self‐concept clarity within a clinical population with bipolar disorder. Additionally, the efficacy of the Identity Disruption Model could be extended outside mood and anxiety disorders, potentially looking at personality disorders where the self‐concept is strongly implicated (Caligor et al., 2015; Kerr et al., 2015).

### Clinical implications

This study was conducted with a non‐clinical community sample and did not use diagnostic measures; therefore, caution must be used in drawing conclusions about the clinical implications of the findings. Nevertheless, evidence supports the conception of psychopathology, including depression and anxiety (Haslam et al., 2012) and hypomania (Meyer & Keller, 2003) as dimensional rather than categorical, which suggests that the observed results are likely to hold in clinical populations. Our results, coupled with those from previous work (Hayward et al., 2020; Wong et al., 2019), suggest that increasing self‐concept clarity might have a direct positive impact on psychological distress, such as depression, anxiety and hypomania. Previous work has demonstrated that it is possible to experimentally manipulate and increase self‐concept clarity and that increases in this factor were linked to improved relationship quality (Lewandowski Jr et al., 2010), which suggests such interventions are plausible. Given that self‐concept stabilizes in adolescence (Habermas & Paha, 2001), which is also a time when emotional and psychological problems are particularly prevalent (Deighton et al., 2019; Kieling et al., 2011) and appear to becoming more common (Bor et al., 2014; Van Droogenbroeck, Spruyt & Keppens, 2018), work targeted at improving self‐concept clarity in children prior to adolescence might be most effective in reducing levels of psychological distress in later life.

## CONCLUSION

Current findings confirm that childhood adversity predicts depression and anxiety directly and indirectly via the parallel mediators of self‐concept clarity and self‐esteem. However, intolerance of uncertainty mediated this link for anxiety but not depression. The current study also provided important novel evidence that self‐concept clarity mediated the influence of childhood adversity on hypomania. Taken together the current findings provide evidence for the identity disruption model, which posits that early aversive experiences disrupt development of personal identity, resulting in an unclear sense of self and greater reliance on external sources of self‐identification, which then promotes psychological distress. Findings suggest that targeted interventions to improve the clarity of the self‐concept could have positive effects on levels of psychopathology.

## AUTHOR CONTRIBUTIONS

**Nathan Ridout:** Conceptualization, methodology, formal analysis, project administration, supervision, validation, and Writing—review and editing. **Lindsey Sharratt:** Conceptualisation, methodology, investigation, formal analysis, data curation and Writing—original draft.

## CONFLICT OF INTEREST STATEMENT

Neither of the authors have any conflicts of interest to report.

## Data Availability

The data that support the findings of this study are available from the corresponding author upon reasonable request.
